# Families of victims of homicide: qualitative study of their experiences with mental health inquiries

**DOI:** 10.1192/bjo.2020.84

**Published:** 2020-09-02

**Authors:** Lillian Ng, Alan F. Merry, Ron Paterson, Sally N. Merry

**Affiliations:** Department of Psychological Medicine, University of Auckland; and Counties Manukau District Health Board, New Zealand; Department of Anaesthesiology, Faculty of Medical and Health Sciences, University of Auckland; and Department of Anaesthesia, Auckland City Hospital, New Zealand; Faculty of Law, University of Auckland; Melbourne Law School, University of Melbourne, Australia; and New Zealand Government Inquiry into Mental Health and Addiction, New Zealand; Department of Psychological Medicine, University of Auckland; Cure Kids Duke Family Chair in Child and Adolescent Mental Health, New Zealand; and Werry Centre for Child and Adolescent Mental Health, New Zealand

**Keywords:** Homicide, victim, inquiries, mental health services, serious incident

## Abstract

**Background:**

Investigations may be undertaken into mental healthcare related homicides to ascertain if lessons can be learned to prevent the chance of recurrence. Families of victims are variably involved in serious incident reviews. Their perspectives on the inquiry process have rarely been studied.

**Aims:**

To explore the experiences of investigative processes from the perspectives of family members of homicide victims killed by a mental health patient to better inform the process of conducting inquiries.

**Method:**

The study design was informed by interpretive description methodology. Semi-structured interviews were conducted with five families whose loved one had been killed by a mental health patient and where there had been a subsequent inquiry process in New Zealand. Data were analysed using an inductive approach.

**Results:**

Families in this study felt excluded, marginalised and disempowered by mental health inquires. The data highlight these families’ perspectives, particularly on the importance of a clear process of inquiry, and of actions by healthcare providers that indicate restorative intent.

**Conclusions:**

Families in this study were united in reporting that they felt excluded from mental health inquiries. We suggest that the inclusion of families’ perspectives should be a key consideration in the conduct of mental health inquiries. There may be benefit from inquiries that communicate a clear process of investigation that reflects restorative intent, acknowledges victims, provides appropriate apologies and gives families opportunities to contribute.

## Background

Families who have experienced the loss of a loved one as a consequence of homicide where the perpetrator was receiving mental healthcare are a unique group whose voices have rarely been sought.^1^ A homicide by a person in receipt of mental healthcare is a serious incident^2^ and investigations of mental healthcare related homicides may give families an opportunity to present the victims’ perspective.^3^ Key principles for investigating serious incidents in healthcare include a process that is open and transparent and an approach that is objective, timely and systems focused.^2^ The purposes of inquiries may be to establish facts, provide an impetus to learn from events leading to the incident,^4^ hold multiple people or systems to account or to reassure the public.^5^

Staff, patients, victims and perpetrators and their families and carers are all affected by homicide. An inquiry provides a means to highlight gaps in systems and processes of care that can result in serious incidents.^6^ Serious incidents include acts or omissions in care that can result in serious injury or unexpected death.^2^ Homicide, the crime of killing a person, is subject to particular scrutiny when the perpetrator had a psychiatric illness as the care of the perpetrator may be retrospectively analysed to determine whether the death could have been prevented.^7^ It is recommended that patients and victims’ families are involved and supported throughout an investigation process.^2^ However, victims of mentally disordered offenders feel isolated and unsupported by healthcare and legal systems.^1,8^

## Types of inquires in New Zealand

In New Zealand, inquiries following mental healthcare related homicide include hospital serious incident reviews (internal and external), coronial inquests and formal complaint procedures. District health boards, responsible for providing mental health services in New Zealand, may conduct a serious incident review. These usually precede coronial inquiries or external inquiries requested by the Director of Mental Health (within the Ministry of Health, the government agency responsible for district health boards) under specific mental health legislation and investigations initiated by a formal complaint to the New Zealand Health and Disability Commissioner.

Information may be shared between different inquiries to avoid duplication and expedite investigations (according to a Memorandum of Understanding between the Office of the Chief Coroner and the Office of the Health and Disability Commissioner, 2016). Unlike the National Confidential Inquiry into Suicide and Homicide by People with Mental Illness,^9^ New Zealand does not hold details of homicides and suicides by people under the care of mental health services in a central repository for clinicians to access, for conducting research or for developing national policies. There is no official guidance to standardise inquiries across the 20 district health boards in New Zealand.^10^

Families of victims of mental healthcare related homicide seek an explanation of what happened and wish to know what improvements will be made to services.^11,12^ Yet such families describe invalidating experiences,^13^ difficulties establishing contact with hospital managers and struggles with obtaining information from mental health providers about investigations of perpetrators’ care.^8^ Healthcare services may not feel the same obligation to disclose information to victims’ families^8^ compared with families whose loved one died as a result of a medical adverse event^14^ and may be uncertain about what information to share with families.^15^

Theoretical approaches to inquiries have been proposed for adverse events in healthcare.^16,17^ Reason's model, used to analyse complex industrial accidents,^18^ has been adapted for use in medical contexts.^16^ This conceptual framework includes consideration of attributes of the patient, team and organisation in relation to the outcome. Mental health inquiry panels have developed systems-based protocols to conduct inquiries, a form of structured analysis that explores contributory technological, psychological, social and human factors to adverse outcomes.^17^ There is limited evidence that investigations with a systems focus are effective in recommending and implementing useful changes to mental health services.^16^

## Aims

Our observation that the views of families were often missing in inquiries led to this study. Inquiries have reported on relationships between healthcare and other systems and how peoples’ actions and choices are influenced by the system within which they are working.^19,20^ The authors’ experiences of working within New Zealand's healthcare system influenced the study design. Our intent was to return findings to the field of practice and present evidence for consideration of potential changes to inquiries that could benefit those involved with them.

In this study we have explored families’ experiences of inquiries related to mental healthcare. Victims of crime who encounter the legal system have a high risk of a negative mental health impact and of re-traumatisation.^21^ There are few studies that specifically document families of victims’ experiences of investigations into the mental healthcare of perpetrators.^21,22^ In principle, including the perspectives of families of patients and victims is good practice for inquiry panels. Yet, families of victims of homicide describe difficulty navigating mental health systems and obtaining information when perpetrators have a psychiatric illness.^8^

This study is part of a larger body of work to investigate the perspectives of various stakeholders in mental healthcare related inquiries in New Zealand, including their expectations of and experiences with the process. Our wider study extends to the views of clinicians, and members of inquiry panels. This article reports on the participation of families of victims in mental healthcare related inquiries, their understanding of an inquiry's purpose, and the support they received. By investigating families’ experiences with mental healthcare related inquiries following a homicide perpetrated by a patient, there is potential to identify how mental health services can better respond to their needs and concerns when conducting reviews of serious incidents.

## Method

### Study design and methodological considerations

The study design was informed by interpretive description,^23–25^ an approach to qualitative research whereby ‘logic derived from the disciplinary orientation’^25^ is applied to analysing a phenomenon. Our research explored families of victims’ experiences of serious incident reviews following mental healthcare related homicide. Our experiences as clinicians, researchers and government policy advisors span systems of general and forensic psychiatry, anaesthesia, law and the safety and quality of healthcare. The primary analysis was conducted by L.N., who is a forensic psychiatrist. Interpretive description was chosen to address the study's question as it enables clinicians to engage with research at the junction of clinical practice. The focus of our research question was the participants’ experiences of district health board inquiries. We presumed that our research question intersected with clinical practice and policy, and that the first author's (L.N.) clinical experience in mental health and forensic services, and with conducting inquiries, would enhance the qualitative analysis undertaken. The process was iterative with immersion and deep engagement with the data (by L.N.) to develop initial codes and facilitate the development of conceptual themes, sharing the analysis with participants for further feedback, and discussing findings with mental health clinicians to broaden perspectives.^26^ To enhance trustworthiness, an independent researcher (a psychologist experienced in qualitative methods) co-coded the data, provided a commentary and contributed to the development of themes over a period of 6 months. This was developed further by disseminating findings to clinicians working in mental health services. In this step, L.N. presented initial findings to clinicians and discussed how these might be received and applied in practice.

### Participants

Participants were families of victims of mental health homicide, that is, a member of their families had been killed by a patient under the care of mental health services in New Zealand. A family may be defined as a group of people that may be made up of partners, children, parents, aunts, uncles, cousins and grandparents. The term ‘under the care of mental health services’ refers to patients formally under the care of a district health board mental health team (secondary services). Additional inclusion criteria were: age 18 years or above; able to give informed written consent; a serious incident review was conducted by a district health board mental health service between 2002 and 2017; and contact with the family did not breach New Zealand privacy legislation. A New Zealand Health and Disability Ethics Committee approved the study, with conditions that participants be recruited via a third party (ethics approval number 17/NTA/228).

Originally, we proposed a purposive sample to access participants and approached two key New Zealand agencies: Victim Support (a non-governmental organisation that supports victims of crime) and the Ministry of Justice (which holds the Victim Notification Register containing details of victims, for the purpose of informing victims about events regarding the offender). Both agencies declined to facilitate entry into the study; the former cited a lack of resources and the latter cited privacy interests. Accordingly, the study used a snowball sample. In total, five participant families were recruited between August 2018 and March 2019. The first participants were recruited via a key informant in mental health advocacy, who recommended a chain of potential respondents. Additional participants were recruited through hospital managers and family advisors based at the district health board mental health services in Auckland, New Zealand, who identified family members of people who had been victims of homicide perpetrated by a mental health patient. Participants who expressed interest in the study were sent an information sheet and a copy of the interview schedule, which was followed up by a telephone call from the first author. Participants gave their written consent to take part in the study.

### Interviews

Before commencing the study, the semi-structured interview schedule (see Appendix) was checked by, discussed with, and approved by a family member of a patient who had died under the care of hospital services (not mental healthcare related). Between July 2018 and March 2019, the first author interviewed all participants. They were invited to bring a support person and asked if they wanted specific cultural support. Interviews began with acknowledging the victim and exploring families’ involvement and understanding of inquiries. Families were also asked to reflect on their experiences of the inquiry process, including the support they received. Interviews were guided by participants’ concerns and adjusted accordingly. Some participants provided written reflections. Interviews were audio-recorded and transcribed verbatim. Following the interviews, participants were offered psychological counselling, funded by the study.

### Analysis

The interview transcripts were returned to the participants to confirm accuracy of the data. Names and locations in the transcripts were de-identified and a code was assigned to each transcript. NVivo was used to store and manage the data. All transcripts were read intensively and primarily coded by the first author, using an inductive approach.^27,28^ Memos, observations, reflections and critical questions were recorded across the data-set. An independent researcher (a psychologist-researcher) co-coded a portion (20%) of the data, peers reviewed the coding process and verified the coding framework.

During the analytic process, codes and memos were checked against the transcripts. As described above, concept themes were developed.^29,30^ The results were presented to mental health clinicians at educational forums in New Zealand and Australia. Verbal and written feedback was incorporated into the development of key themes. The themes were further developed in discussion with an external supervisor (a psychiatrist) of the study. This step may be described as the ‘thoughtful clinician test’,^25^ whereby expert practitioners are considered a ‘collateral data source’ to critically reflect on perspectives of the phenomena. This formal relationship also acknowledged the sensitive and emotionally demanding nature of the research.^31^

## Results

Five families of homicide victims participated in separate interviews and one family provided additional written reflections. There were a total of nine participants in the study involved with four different New Zealand district health boards that included family members who were parents, siblings, sons and daughters of the victims (Table 1). One family included parents of a mental health patient who was killed by another patient. The remaining family members had little or no experience of mental health services as consumers. The families were involved in a range of inquiries, including hospital serious incident reviews (internal and external), coronial inquests and formal complaint procedures. All participants accepted post-interview counselling sessions. Three elements of a good inquiry emerged from the data: understanding the perspectives of families of victims, communicating a clear process of inquiry, and acting with restorative intent. Quotations illustrating these themes are presented (see also the supplementary data, available at https://doi.org/10.1192/bjo.2020.84).

**Table 1:** Description of participants

Case	Participants, *n*	Brief description of participants
1	2	Brother and sister-in-law of victim. First point of contact to invite families from cases 2, 3 and 4. Victim was killed by a mental health patient who was found not guilty by reason of insanity. Two subsequent inquiries of the patient's care: (a) by the district health board and (b) by the New Zealand Health and Disability Commissioner.
2	1	Daughter of victim. Victim was killed by a mental health patient who was found not guilty by reason of insanity. The case was part of an external inquiry of other mental healthcare related homicides in the same region.
3	3	Son, daughter-in-law and daughter of victim. Victim was killed by a mental health patient who was found not guilty by reason of insanity. Two subsequent inquiries of the patient's care: (a) by the district health board and (b) by the coroner.
4	1	Mother of victim. Victim was killed by a mental health patient who was found not guilty by reason of insanity. Two subsequent inquiries of the patient's care: (a) by the district health board and (b) by the coroner.
5	2	Mother and father of victim. Recruited by family advisor at a secondary mental health service. Victim was a mental health patient, killed by another mental health patient who was convicted and imprisoned. The case was a serious incident reviewed by the district health board.

### Understanding the perspectives of families of victims

The families in the study expressed initial bewilderment that their loved one had been killed by a mental health patient and disbelief when they felt contact with hospital authorities lacked empathy for their loss and circumstances. Their experiences of mental health inquiries into the care of the perpetrator were marked by exclusion, marginalisation and disempowerment. Several participants spoke of their disappointment that the victim and their needs were not enquired into.

‘[Victim] wasn't at the forefront of his own death. In a way he was collateral damage, he was secondary to their thoughts. They were more worried about their reputation and what they did, or didn't do, what was missed out was health and well-being and recovery. They weren't concerned about ours, or how he died.’ (Family 1)

These participants’ concerns continued as they described their sense of exclusion from the inquiry process. These families felt angry at being ‘shut out’ and some described their perception of a lack of respect shown to them by hospital providers. This was more prominent when participants discovered information second-hand, from the media.

‘The way I found out was pretty much newspaper articles, yeah literally. So all my information was newspaper articles and I didn't find that acceptable.’ (Family 2)

The sense of exclusion was compounded by feeling marginalised from the inquiry process. Some families met with hospital representatives and spoke about their concerns that district health boards focused on being defensive, rather than empathetic. These participants expected an acknowledgement of loss of their family member. With the exception of one family, condolences were absent.

‘I was expecting them to say “look we're really sorry about that” or “this has happened to [victim]”. I was numb at that stage, I just walked out. My cousin was behind me and I did hear him say “I can't believe you people just sat there and didn't say sorry”. Then we just left and that was all there was to it.’ (Family 3)

The participants’ attempts to access information from hospital authorities were frequently unsuccessful. They were told the information could not be divulged because of the need to protect the perpetrators’ privacy. Several families requested basic details relating to the perpetrator, or inquiry. They emphasised that they wanted information about public safety, not confidential medical information. Over time, these family members continued to have unanswered questions about the perpetrator's mental healthcare.

‘It [an inquiry] could have given me some closure instead of having all these questions and no one to answer them. There are always so many questions. There was just no one to answer or give me an insight of why it happened and how it happened.’ (Family 2)

These families described their disempowerment and the cumulative psychological stress that arose from their attempts to obtain information.

‘People are absolutely appalled at the way [we've] been treated and the length of time it's gone on. The emotional trauma that it has caused us. It's been absolutely horrendous.’ (Family 4)

The participants in the study spoke of difficult emotions in waiting for various inquiry processes to be completed over a period of months and, in some cases, years. Some described the double pain of loss and injustice as perpetrators were found legally insane. In these cases, families highlighted they felt further excluded and isolated, with few avenues of recourse to address their concerns about process.

### Communicating a clear process of inquiry

All the families in the study referred to a serious void in communication from mental health services. These families described a paucity of information about the inquiry process and its purpose. One family articulated their response to the lack of process:

‘There was no process, that's what's most frustrating, that's why I feel like I just want to sue [the hospital]. Hurt them in some way so that it makes them have a process. I just try to forget about it, I just bury it deep and don't talk about it or think about it, I don't ever.’ (Family 3)

These participants considered essential elements in communicating process to include an open invitation to participate; a verbal and written explanation about the purpose of the inquiry and how the case would be investigated; and sharing the findings and recommendations with them. Several highlighted that written information may have helped them understand what the process of a district health board inquiry entailed:

‘I don't know whether it was because we were caught up in the court case or whether it was too fresh. We didn't really understand what the review process was, who would be part of it, what our involvement would be for opportunity for input.’ (Family 5)

Several families reported that their contact with district health boards during and following inquiries was harmful. Some examples were given: a brief summary report in their mailbox; being made aware of an inquiry after it had been completed; and not receiving findings or feedback following the conclusion of an inquiry. They spoke of lost opportunities to make sense of events in the narrative of the victim, identify gaps in mental health service care of the perpetrator and contribute to improving services.

‘If [we] could have been told about those results and the processes that they put in place to actually learn from what happened, I believe that would have made a difference, it might not have resolved all the anger or frustrations but it would have been a big step in the healing.’ (Family 3)

Several families expressed their feelings of frustration and mistrust of hospital providers over time. They viewed access to support and advocacy, as important in understanding their rights, and what they could expect from mental health services. As they progressed with other independent coronial or complaint-related reviews, they became concerned about transparency in the conduct of mental health inquiries.

'Transparency is a tough one to achieve when [the medical profession] is self-regulating and does its own review. There needs to be an independent review and you can't rely on the [hospital] to do its own review because it just doesn't work. You're not going to get people crucifying themselves for their own performance.’ (Family 3)

Despite their concerns, the participants described a role for mental health inquiries in answering specific questions about the perpetrators’ care. This was something often addressed less effectively in legal inquiry processes. Their experiences of coronial inquests were mixed as in some cases a coroner's review occurred many years after the homicide. In general, the participants spoke more positively about coronial inquiries. Several participants described the difficulty in understanding processes and the links between different inquiries and proceedings after they had been completed.

‘With the benefit of hindsight, I would have considered taking legal advice to better understand options for recourse… the process used to incarcerate [perpetrator] under the Mental Health Act, the process of review of [perpetrator]'s imprisonment and/or release…and civil proceedings against [perpetrator] or the [hospital] for the emotional harm they have caused us.’ (Family 3)

Several families in the study stated that the provision of clear information at the outset may have helped them understand what they could expect from an investigation.

‘[Victim] died and then that was it. If we'd got something back from the report, we would have got some sense of closure.’ (Family 5)

These participants were left with unanswered questions related to the perpetrators’ care and recommendations and changes that would be made to services as a result of an inquiry.

### Acting with restorative intent

The participants in this study spoke of their grief in losing their family member and described actions that could promote healing. Actions that demonstrated restorative intent emerged as an important attribute of the inquiry process: for district health boards to demonstrate a sincere intent to engage with them; to acknowledge the victim, apologise to the family and convey a commitment to undertake an inquiry with integrity. For several participants, the lack of acknowledgement of their loss and needs perpetuated their grief:

‘No one wants to acknowledge that you had a stake in the whole thing, an opinion, maybe a solution or a point of view.’ (Family 1)

‘One thing that would have really helped was an acknowledgement and some form of apology [from mental health services]. We've never had that…just recognising a life was lost and that person was here. They need to realise it's people they are dealing with.’ (Family 4)

For one participant, the humanity of the victim was not acknowledged until many years later at a coronial inquest:

‘It [coronial inquest] was the first time that [victim] had ever been thought of as a person.’ (Family 4)

Several families wanted to contribute to district health board inquiries to enable mental health services to improve care, and for their perspectives to be recognised and valued.

‘I do see something coming out of the process if I was involved as long as I had an opportunity, not a right. An opportunity to query, or challenge or respond, not just get the findings…prior to that just saying, these are our preliminary findings, we value your input, it could be valuable to us. At least it would give you a sense that you are actually contributing to the process getting better.’ (Family 3)

These families wanted to see evidence that meaningful learning had taken place to help prevent similar mistakes in the future.

‘I think confirmation a lesson has been learnt. If you had feedback from an inquiry to say we've learnt this lesson, we've amended our process. Thank you for your input. That would make me feel okay, something good has come from this.’ (Family 3)

Several participants related concerns that inquiries were not disseminated to mental health services nationally and that district health boards did not learn from inquiries as inquiry recommendations were not formally enforced.

‘All they are is recommendations, this is a real “stuff you” to the victims, not only did [the hospital] ignore them, they also sent a letter to the coroner telling him he was wrong…That just piles contempt on top of contempt.’ (Family 3)

‘It's all very well putting something on a piece of paper…unless you actually act and implement change, then to be honest the review's a total waste of time.’ (Family 4)

Several participants spoke of feeling aggrieved and re-traumatised by denial of accountability by mental health services. One family received a mandated apology many months later following an inquiry. These actions were perceived by participants as harmful, insincere and disrespectful to the memory of their loved one.

‘We've had to fight for everything. When I say fight I mean Official Information Act, Ombudsman, everything, they would not give us anything without making us fight for it. That's going on behind the scenes while you're trying to go through a court process into a murder. You're not only fighting the justice system, you're fighting the Ministry of Health, and individual [hospital service] for stuff that should be available pretty early, as of right.’ (Family 1)

Several participants who had negative encounters with mental health services proactively sought further information from mental health services and accountability from governmental bodies. Some escalated their concerns to formal complaint procedures and became advocates for families with similar circumstances.

## Discussion

In this exploratory study, we examined the experiences of mental health inquiries from the perspective of a small number of families of victims of homicide in New Zealand. Their complex experiences suggest a strong sense of exclusion and disempowerment following the death of their family member. We were moved by the depth of feeling of these participants over time and their unresolved questions despite the inquiry processes. Our results suggest that these families sought to engage with district health boards during these inquiries specifically to better understand the circumstances of their family member's death and what changes could be made to secondary mental health services to prevent a similar death in the future. They received limited information and little or no formal support from mental health services. First steps in promoting healing would presumably include an acknowledgement of loss by district health boards and the communication of a clear process but these steps were typically missed out.^1,32^

Although this study has been carried out in a small population in New Zealand, there are similar processes for inquiries internationally.^33^ The UK's National Health Service Serious Incident Framework notes a central premise of an investigation is to ensure learning is prioritised to prevent the likelihood of similar incidents occurring in the future.^2^ A review of investigations of the deaths of patients concluded that many carers and families do not experience healthcare providers as open and transparent.^8^ Secondary victims, such as families of victims of mentally disordered offenders, who may have encounters with legal systems in these processes may be exposed to psychological risks.^13^ Many do not have access to an advocate and feel unsupported.^8^ The present study contributes to an ongoing dialogue about the responsibility for creating a safe psychological climate for families of victims involved with mental health inquiries.

In practice, investigations vary, as does the communication of findings to families.^34^ The participants in this study were involved with various medico-legal proceedings in the time that had elapsed since their family member was killed. The lengthy time frames meant it was not possible to narrow the focus of the study to one type of inquiry, for example, the hospital serious incident review of mental healthcare of the perpetrators. The study reveals the difficult experiences of families of victims in navigating multiple processes of inquiry. Not all were involved with the hospital serious incident review process. However, they emphasised this investigation as particularly important as a source of information about the events leading up to the death of their family member and the mental healthcare the perpetrator received.

This study highlights the potential for inquiries to have a restorative function,^14,15^ in addition to that of documenting and interpreting events.^5,16^ This is exemplified by the participants’ experiences of exclusion from inquiries that led some to pursue information and accountability through a wider system, including formal complaint and legal processes.

It is also reasonable for the family of a victim to expect an explanation, acknowledgement of^35^ and apologies for any failings in care and communication in a timely and sensitive manner.^1,36,37^ This study has highlighted the potential neglect of families during inquiry processes in New Zealand. We postulate that this is likely to be true elsewhere, as well. We suggest that healthcare and mental health service providers should consider families of victims as key stakeholders, obtain their perspectives and consider making formal access to support available to them. The focus on experiences of families of homicide victims as a group with distinct needs^8^ could assist with debriefing and educating front-line clinicians. Our findings may encourage members of inquiry panels to view the inclusion of families of victims as a vital step in the process and one which may attenuate these families’ emotional distress.^21^ Communicating a clear process and the findings of the inquiry may help a little to mitigate negative consequences of loss as a consequence of homicide.^1^

### Strengths and limitations

The rapport built with participants in initial contact and sensitive interviews and the capture of depth and richness of their experiences are strengths of this study. Transferability of the findings to other contexts, may be limited by the small sample size, the methodology and analytic process. These data represent the experiences of New Zealand families who chose to participate in this study but cannot be assumed to represent those of all families of victims. The participants in this study may have felt most strongly about their experience of inquiries, or found the process particularly distressing. The findings may guide reflection on approaches to deriving wider purposes and meanings from inquiries of this type. The dissemination of findings to clinicians working in mental health services revealed practical tensions in responding to families of victims at an individual level and developing policy responses to families of victims at local service and wider system levels. Although clinicians identified with the participants’ experiences, their capacity to effect changes in inquiry practice is limited to their individual contexts and involvement in inquiries.

### Future research

Family members of victims of homicide are one stakeholder group in mental healthcare related inquiries, and those who participated were a small subgroup of the whole, limited to New Zealand. However, their feedback was common to all, and in line with concerns raised in the literature. Investigating whether these findings apply more generally is important. Another important group to consider is the family members of patients who have been perpetrators of homicide. Families of victims and perpetrators are both groups that are difficult to access, as their private information is held by gatekeepers. Understanding the impact of inquiries on clinicians^38,39^ and the perspectives of those conducting inquiries would also help guide the development of a more tailored framework for conducting inquiries into serious mental health incidents, and one that can better address the needs of families.^16,40^

### Implications

The data in this study have highlighted a gap in the way inquiries are conducted. Families in this study were united in reporting that they felt excluded from mental health inquiries. We suggest that perspectives of families of mental health related homicide should be a key consideration in the conduct of mental health inquiries. There may be benefit from inquiries that communicate a clear process of investigation that reflects restorative intent, acknowledges victims, provides appropriate apologies and gives families opportunities to contribute.

## Data Availability

The authors report direct access to the study data. Access to transcripts of interviews with participants is ongoing and stored in accordance with New Zealand ethics committee guidelines. The analysed data is provided and can be accessed via the supplementary data.

## Supplementary material

For supplementary material accompanying this paper visit http://dx.doi.org/10.1192/bjo.2020.84. click here to view supplementary material

## Appendix

### Participant interview schedule

**Table:**

1) Would you mind telling me about your family member. What was he/she like?
2) Would you mind sharing with me your involvement with the inquiry after the incident involving your family member?
3) Did you feel that your views were included during the process of the inquiry? Did you feel that your views were respected during the process of the inquiry?
4) What opportunity were you given to participate? If not, what input would you have wanted to provide to the inquiry?
5) What was your understanding of the purpose of the inquiry?
6) What information was given to you about the process of the inquiry? If not, what information would you have wanted?
7) What form of communication did you receive about the inquiry?
8) What did the process of inquiry provide for you and your family? What feelings did the process of inquiry bring up?
9) Was the inquiry conducted in a timely manner? If not, what timeframe would have been acceptable?
10) What sort of feedback was given to you after the report was published? Were you aware of the findings and recommendations? What way would you have liked to receive the report findings?
11) Did you think that the inquiry achieved its purpose? Did you gain a clearer understanding of what happened? Did you feel someone was held to account? What did you think professionals could learn? Is there something that mental health services could do differently?
12) Were you aware of the recommendations from the inquiry report? Were you aware of a timeline for implementing these recommendations? Who should monitor/follow up these recommendations?
13) Would you mind sharing your views on publishing findings from inquiries? What are your views about making a de-identified inquiry available in the public domain?
14) Can you tell me how you felt about the process?
15) What sort of support did you receive during the process of inquiry? Who did you receive support from? If no support, what support would you have liked to have had?
16) Did the process of inquiry provide you with any sense of resolution?
17) What difference did the inquiry make to you and your family?
18) What sorts of things were you seeking from the inquiry?
19) Would you mind sharing with me what you believe is important in an inquiry?
