# Supplementary material for: Families of victims of homicide: qualitative study of their experiences with mental health inquiries
Source: BJPsych Open. 2020 Sep 2;6(5):e100. doi: 10.1192/bjo.2020.84 (PMC7488330; doi:10.1192/bjo.2020.84)
Supplement: Supplementary file 1 [file bjosup.zip › S2056472420000848sup001.docx]

| 1. **Families perspectives** |
| --- |
| Legislation prohibits us really from having rights, the patient’s rights supersede any victim rights. They cite patient confidentiality and privacy as a reason to deny us even basic information about a mentally ill offender, which again re-victimises us. (Family 1) |
| I was really angry. [The psychiatrist] was forced to apologise, which he did, but it meant nothing because it was forced. By his statement saying that he still felt that he was unjustly targeted and he did nothing wrong, means that his apology was insincere. He wasn’t made accountable as such. (Family 1) |
| [Other families] have felt very under-valued and disempowered by that whole process [series of inquiries]. (Family 1) |
| It wasn’t really something we were so conscious about at the time because we were going through the court process, we had all that distraction. The Health Board thing wasn’t really in our sights at that time, we were fending off inquiries from the media. Trying to get our heads around the whole judicial process, which was equally as appalling as the District Health Board treatment of us, to be frank. (Family 1) |
| It’s quite heartbreaking and frustrating that you don’t get that opportunity and you're just ignored…[the DHB] should be doing harder research into finding [family members]…it was quite upsetting that I've been pushed to the side and forgotten about, no one really actually asked me for my opinion. (Family 2) |
| The fact that [media] knew more than I did was kinda upsetting. (Family 2) |
| It has done some damage because I've had the built up feelings, emotions, and all of that…I was just kinda left alone. (Family 2) |
| I didn't even know about inquiries. Like knowing that an inquiry is done would even be nice. (Family 2) |
| I think [knowing about the inquiry] would make a difference, I could at least offload how I feel and have an input. (Family 2) |
| To be listened to and felt like my opinion did count in the inquiry. It is kinda hard just being ignored and not even feel like someone’s listening to you or cares about your feelings. It would have been nice if they had made contact… to meet with me and talk about my experience. (Family 2) |
| The first meeting we had we just sat there dumbfounded about what was being said. We were still in shock and just absorbing what happened. (Family 3) |
| That’s what frustrating, and I just try to forget about it, I just bury it deep and don’t talk about it or think about it, I don’t ever, because it's just frustrating. That’s what eats me up is it's frustrating, there's just nothing, there’s no process. (Family 3) |
| I feel the DHB has treated me, my family, the local community and my father’s memory with utter contempt. (Family 3) |
| They lie, they just absolutely lie, and it just adds to the frustration and the sense of contempt. (Family 3) |
| Oh it just compounds on numerous levels. (Family 3) |
| I If you had feedback from an inquiry to say our process is just this, because of what has happened we’ve now learnt this lesson so we’ve amended our process so now we take in to account this. Thank you for your input. That would make me feel at least okay, something good has come from this. From my experience that just doesn’t happen. (Family 3) |
| I cannot express who insulting and bewildering I find it that a government organisation can take actions like this that place the local community…at risk. I feel the [named] DHB has treated me, my family, the local community and my father’s memory with utter contempt. (Family 3) |
| That strong one for me is being acknowledged and being invited into that process [DHB inquiry]. They need to work out a way that they're gonna do that because a lot of families probably wouldn’t be necessarily ready for it at that stage. Those communication doors need to be starting to open. Probably a written thing is probably the best way to start with. (Family 4) |
| I think that when we’re shut out and we don’t understand [why] we’re shut out. It just makes you angry. I think that you need to be part of that process. (Family 4) |
| Humanity I think more than anything. They need to understand that things need to be improved. There needs to be communication and people need to be treated properly and thought of. (Family 4) |
| [It’s important] that [the victim] is being considered because she was never considered. That was the worst thing, I think that was just the ultimate. (Family 4) |
| [The victim] didn’t have that voice, she was never, ever given that chance. She became irrelevant in the process. It's been really important for us to be able to do that and speak up for her…the first time that we’d ever really been able to actually relate [the victim’s] side of it was [at the coronial inquest]. That was [many] years down the track. (Family 4) |
| In a sense [the coronial inquiry] was the first time [the victim] had ever been thought of as a person. The Coroner was very good…he did humanise her. It answered a lot of things. It did open up a can of worms because it opened up more questions. (Family 4) |
| To be aware that [the DHB inquiry] was going on in the first-place and not find out in the media. To be invited to participate would have been courteous, to be explained the process and the terms of reference. Just anything apart from zero, I mean anything above what we got would have been a bonus, but to get absolutely nothing, it’s not acceptable. (Family 4) |
| I've talked to people here and they are absolutely appalled at the way that the whole thing’s been treated and the length of time that it's gone on. The emotional trauma that it has caused us. It's been absolutely horrendous. It’s not just us, it's the extended family and friends. (Family 4) |
| We weren’t even aware of mental health input in the very beginning. It wasn’t until after [the victim] was killed that we even knew that there had been issues…we didn’t even know there were any mental health issues, even up to that stage. That is how much we were kept out of it. I do believe that that’s something that we should have been privy to at that stage, because [the victim] had been murdered. I think we should have been at least given some guidance (Family 4). |
| The families need to be involved in [the DHB] side of it. At that time when the grief is really, really raw, I think that's actually a good time. Although it's probably isn’t for everybody, for us it would have helped. We could have dealt with it then and perhaps the lengthy process would have been shortened because we had to wait [many] years by the time we got the Coroner’s report. (Family 4) |
| With the passage of time there’s a sense of it would have been nice to have had some sort of follow-up with the DHB in time, because we were so busy after [the victim’s] death dealing with loss…the practical things and the court case. Now that we’ve had a chance to catch our breath, even an open invitation whenever you felt like you wanted to meet to have a discussion. (Family 5) |
| 1. **Process** |
| The whole process is just absent from my experience. But if you're going to have a process from a victim point of view, I think that would be critical, is some feedback to the victims that something has been learnt. (Family 1) |
| We didn't know there was the serious incident [report], didn't know about that. The external investigation had been done and dusted by the time we were all aware of it. The only reason I knew about it, it was in the media that they had had it but that process had already been completed. So there was no chance of any involvement in that. (Family 1) |
| Now, realising we’d been excluded from the whole process, a [coronial] inquest would have been an opportunity for us to confront [the district health board] with some of these questions. (Family 1) |
| What could be worse than what’s already happened? Being given the opportunity is far better than being denied the opportunity. (Family 1) |
| There wasn’t a process. It wasn’t any involvement [from the family] because the central figure in that was [the perpetrator] not [the victim]. (Family 1) |
| There’s nothing in the report that suggest they did speak to [other members of the family]. (Family 1) |
| [I would have wanted to receive the report] personally…With an explanation, go through it, explain the terminology and how they came to the [conclusion]. (Family 1) |
| Some opportunity to have input. We can’t obviously have input without knowing the background. We’re a third party. It’s not like we were [the perpetrator]’s family that knew his history. (Family 1) |
| Just be included would be helpful… just out of courtesy, out of respect for what has happened, you should be included. (Family 1) |
| The more I've dealt with the Ministry of Health, and DHB’s, it’s quite clear that they don’t want you to be involved. (Family 1) |
| The harm’s been done. The harm was done on the [date of the murder], you know, they can’t do anymore harm. All they can do is stop it happening from someone else and [not] to use an excuse as to not involve or contact somebody. It’s just justifying why they don’t contact them, or ask them to be involved. (Family 1) |
| It’s been a fight the whole time. No one has wanted to give us any information… the whole health system right through the judicial process, through to the coroner’s decline of having an inquest. You're like a leper, you're being shunned the whole way through. No one wants to acknowledge that you had a stake in the whole, an opinion, maybe a solution, or a point of view. (Family 1) |
| When you're a key stakeholder in a tragedy like that it should be a no brainer, it should be on the top of the list of people that you invite to be involved in such a process. But currently you're not even on the list. (Family 1) |
| The longer we’ve been involved the more we have been excluded. (Family 1) |
| They're making assumptions on behalf of people, they refer to key stakeholders, not one of those stakeholders was a victim of a mentally ill person. Yet they’ve produced that document with all these recommendations without speaking to somebody that it affects directly. They’ve talked to psychiatrists, lawyers, judges, health professionals and not one victim. (Family 1) |
| There’s a lack of transparency. A lack of transparency rings alarm bells for me, cos as to why, you know I'm directly involved. When my brother died I should have been party to all the information, not just what they decide to let me see. (Family 1) |
| The system, processes, and legislation have made [the victim’s] death worse and harder to deal with. Because they’ve excluded us from the whole thing. As bad as that was, they’ve just put the knife in and twisted it just to make it worse. (Family 1) |
| The coroner doesn't have a vested interest in the outcome…So there’s a bit of objectivity, independence. Perhaps some questions that might be asked that wouldn't have been asked at the DHB inquiry because of the coroner’s lack of knowledge of how the process works. (Family 1) |
| I don’t think there should be the separate agencies, I think there should be a one stop shop…Like the Police Conduct Authority or something like an independent body that investigates homicides or suicides. I think there’s a lot of duplication and waste of people’s time. Disruption to people’s lives by having so many different agencies carrying out their own investigations, where it could be just done in one go. (Family 1) |
| The way I found out was pretty much newspaper articles, yeah literally. So all my information was newspaper articles and I didn't find that acceptable. (Family 2) |
| We didn't get involved, me and my mother, because we were from a previous relationship…so we were kinda just pushed to the side and kept in the dark about everything. (Family 2) |
| I should be involved and considered. The reason they [the DHB] didn't actually talk to me is because everyone refused to give out my details to protect me. But I actually wanted to speak out and be heard about it. So yeah I didn't have involvement til a couple of years ago, and that was about 2016 when I started speaking to [named newspaper] about it, so yeah (Family 2). |
| The fact that [the DHB] don’t actually involve the families, I get it, like they're trying to protect the family and not hurt them by questioning them. But they're not getting their opinion. If I can have a little voice and make a change, then I'm all for it. (Family 2) |
| [The media] started involving me. Letting me know that the DHB was doing a review on their mental health system, which I was all for. I gave them my opinion, that’s when I started getting involved, I was quite happy that they had done the investigation. (Family 2) |
| I wish it had have been the DHB that had involved [me] in the inquiry…rather than left out again. (Family 2) |
| It would have still been nice, even if my mum was notified and she told me. But we both weren't contacted or informed of any proceedings. (Family 2) |
| I think close family should be included, children, the partner of the person at the time, if they have a child from another relationship, that family should be included… pretty much first-hand people should be included in the inquiry. (Family 2) |
| It should be communicated in a way I understand, and if I don’t understand I have the chance to ask for it to be further explained. Everyone gets a written copy of the final results so they can keep that for their documents. It offers a bit of closure to the victims, which I've never gotten. It would be nice if we were actually more involved than kind of being shut off and kept in the dark. (Family 2) |
| [Since the victim was killed] I've had no communication at all from any services. (Family 2) |
| Probably written information, but also the opportunity to contact someone if you don’t fully understand. (Family 2) |
| There was definitely a lack of information and someone pointing out what’s the next step in this procedure. Because I assumed, especially, well [the victim’s] murder was the first one I’d heard about a mental patient or taken notice of. I assumed the DHB’s have quite a grasp on how to minimise their exposure to any negative feedback. (Family 3) |
| You’re trying to negotiate, to navigate what’s happening…I didn’t understand the process. (Family 3) |
| I feel the DHB has treated my family with utter contempt. (Family 3) |
| We were just lost in the system. The mental health side we could have really, really done with some help on that side. Like I said to gain understanding on the processes ‘cos we actually didn’t understand a single thing. A lot of stuff that we were never informed of…it was only through reading in some court documents and we were like, what and when did this happen? (Family 3) |
| The coronial inquiry is where we got to ask questions, we were unprepared for that. (Family 3) |
| If there was a good process the victims would be involved in that process. The findings as much as they could be would be made public. But a DHB review, the medical profession is self-regulating. So there needs to be an independent review and you can’t rely on the DHB to do its own review because it just doesn’t work. You’re not going to get people crucifying themselves for their own performance. (Family 3) |
| [Communication] from the DHB, none. [From] the Coronial inquiry we got a letter back saying yes we were going to have one…it was our chance to ask questions. (Family 3) |
| I would have liked to have been involved [in the DHB inquiry] and had some input in to the process around figuring out what went wrong and why. Because I would probably be asking exactly the same questions as whoever the clinical manager is. Go down to the person at the coalface and say right give us your report, what happened? Cos you know the victims are gonna want to know what happened. (Family 3) |
| The coronial inquiry was the only time we got to ask questions because apart from that the [named] DHB just told us nothing. At least we got to ask questions of someone. In hindsight they weren’t the right people to ask questions to. (Family 3) |
| I asked the Coroner “do I need to lawyer up for this?” and he said no, this is your opportunity to ask questions. In hindsight, I didn’t know who I was going to be asking questions of. If I’d known what was about to happen I would have asked for the CAT [crisis mental health] team to be put up on the stand. Because the one question I’ve never got to ask is whoever made the decision to leave [name of perpetrator] in the public [domain], they’re the ones that should be answering the questions. (Family 3) |
| There's no one because the person doesn’t go through the criminal system, they go in to the mental health system. The mental health system has these walls of secrecy that victims are not privy to so you get left out of the loop. (Family 3) |
| If someone is there to guide [families] at least they'd walk away, say at least there was a process happening and something was learnt. I would think that would be the best outcome from the victim’s point of view. (Family 3) |
| I didn’t even know about [the DHB inquiry] until the Coroner’s inquest, then we were told. We’ve never been told anything. We always feel like we’re like mushrooms, kept in the dark. That a lot of stuff we actually read in the newspaper before we get told anything. (Family 4) |
| Poor communication. In fact it's only ever been instigated when we’ve tried to find things out, or to try and get information, and we’ve been shut down just everywhere we go. It's very, very lacking. In fact I would say non-existent. (Family 4) |
| All I can speak from is what’s happened to us. I have talked to other people in they agree that there’s nothing [communication from the DHB]. I guess we’re not the only ones, but there is nothing. It’s really, really bad. (Family 4) |
| I couldn’t even actually really tell you really what [the DHB inquiry’s] about, because there was very little information. (Family 4) |
| I guess [we wanted] mostly just answers. Answers that probably should have been available to us and information that should have been there. We shouldn’t have go to so much effort to try and find out this stuff. Even to this day they shut us down, and even after everything that's come out of the Coroner’s inquest, [the DHB] still are trying to cover things over. (Family 4) |
| Everything [in the coronial court] was a surprise because we didn’t really have the opportunity [to participate]. The only participation that [husband] and I had was reading out our statements that we’d done for the Police. We couldn’t view any opinions, we couldn’t ask anything. Even then we didn’t feel like we were really part of the process. (Family 4) |
| For closure. For all we know there may be things in the review that we totally disagree with. We haven’t read [the final DHB inquiry report]. (Family 4) |
| We constantly email and ask for updates and where are you on this and stuff like that. A lot of people won’t do that, they give up, it is tiring, it does suck the life out of you. You've just gotta constantly be at it. But we shouldn’t have to be like that. We should be able to have faith in knowing that people are really going to take this seriously. And they don’t want it to happen again or whatever. I mean that's what the whole process is about surely. And I think that's what’s really frustrating. (Family 4) |
| I think that we’d like the opportunity [to participate]. They can't just decide for us. If they gave us the opportunity that would be nice, because we could easily say no, we’re fine, or yes we would like to. (Family 4) |
| I think it gets a bit blurry when you’re dealing with such horrific sort of things. Yeah, you sort of go a bit numb to protect yourself don’t you. (Family 5) |
| I would have liked, once the court case was all over, maybe then an inquiry or review. So when we’re a bit more together I can ask them [about] the processes. (Family 5) |
| Having more information about the review process would have been better. I don’t know whether it was because we were caught up in the court case or whether it was too fresh. We didn’t really understand what the review process was, who would be part of it, what our involvement would be or opportunity for input. We didn’t really realise if our interview with [family liaison person] was our only input, I don’t know whether we were clear about that. (Family 5) |
| I think a bit of time….send out some information that [the DHB is] prior to having the review. What the process would involve… getting feedback and someone actually coming out, so that it's personal. (Family 5) |
| In hindsight we should have understood more about what that review was and what the process was. The fact that we didn’t chase it, probably indicates that we didn’t put a lot of importance on it. Maybe that's because we didn’t necessarily have any concerns about the DHB’s part in the whole event. (Family 5) |
| 1. **Restorative intent** |
| Just regular updates from being informed that there was an inquiry going to take place, your invitation to be a part of it. And how that would look, your participation. Who else is going to be taking part and what their roles are, how they were involved? Just as the process proceeds, to be informed of where it’s up to. And we’ve conducted our interviews, we are now going to sit down and do a draft report. And we will provide it for your comments, you know? (Family 1) |
| If you're denying people information you're disempowering them, the person doesn't know how much other information is being withheld from them. The more information you can pass on the better. (Family 1) |
| [It sounds like you’ve still got some questions?] Yeah I do, because I have just been ignored for years and I've never had the chance to speak to a professional who dealt with his situation, who dealt with his case. (Family 1) |
| How do you arrive at that decision, a professional clinician with years of experience? Please justify how you came to that conclusion especially after all the red flags that were raised. That’s what I'd like to know. (Family 1) |
| I would have liked some answers. I would like to know why a patient who had threatened to kill four people, including psychiatrists, indicating that they wanted to kill somebody on their final admission to the clinic, someone who was not medication compliant, who was using alcohol while in the unit, was discharged with just a prescription. No fixed abode, sent on their merry way, when only weeks before they were discussing sending him to the [forensic] clinic. (Family 1) |
| Certainly no resolution [from the formal complaint process]. All it did was give us more information about what had transpired prior to [the victim’s] death. I think that was the only sort of positive thing. There was no closure, or resolution, or anything from the report. (Family 1) |
| [The formal complaint] provided an insight into the background of what preceded [the victim’s] death…but it was really just a document that gave us more detail…there wasn’t a lot of substance at the end of it… breached code four…back to business as usual. (Family 1) |
| Now, realising we’d been excluded from the whole process, an inquest would have been an opportunity for us to actually confront some of these people with some of these questions. (Family 1) |
| I would like to get their opinion on why he was treated like that and why he wasn’t treated a lot worse than what he was. In my eyes he got let off quite easily and I disagree with that, he should have been put into jail. It would be nice to actually talk to the DHB and [ask] well why? (Family 2) |
| [An inquiry] should be communicated in a way I understand, and if I don’t understand it I have the chance to ask for it to be further explained. Everyone gets a written copy of the final results so they can keep that for their documents. It offers a bit of closure to the victims, which I've never gotten. It would be nice if we were actually more involved than kind of being shut off and kept in the dark. (Family 2) |
| It leaves unresolved issues. It is something that hasn’t been dealt with…The process in itself caused harm in the sense of things not being dealt with, the lack of information, if there is a process there was no information regarding that process. And that has meant that I would suggest that [my family members] feelings haven’t necessarily been dealt with because there was no information about the process and if that process ever had a resolution. (Family 3) |
| Whether the DHB actually ever did its own independent inquiry and came up with results if [we] could have been told about those results and the processes that they put in place to actually learn from what happened I believe that would have made a difference. It might not have resolved all the anger or all the frustrations or whatever but at least it would have been a big step in the healing process. (Family 3) |
| For the process to be better, the DHB should at least try and appear to be less defensive and more factual, it just seemed they were defensive. Like they were starting off on the back foot and they just went hell for leather with we did nothing wrong. So if the process was to be better they would be a bit more forthcoming with what went wrong and then why. (Family 3) |
| Absolutely woeful. So the coroner made that recommendation…you need to review your systems and you need to keep the family informed. I then get this letter from [lawyer] who are Office of the Crown Solicitor, the DHB has now engaged a lawyer who basically refutes the Coroners findings That letter to me is just a big stuff you from the DHB to the victims of [father]. Because the Coroner tells them that they’re remiss in the treatment of [the perpetrator]. (Family 3) |
| My biggest regret is that I didn’t get to ask the right questions of the right people. There were probably two opportunities to do that and one was the coronial inquiry…the other was at the DHB level…If they're gonna conduct a review, get the victims involved. Their input from a clinical point of view was minimal, marginal, probably even irrelevant. It just feels that your loved one didn’t die just absolutely for no reason. Then the state is burying it…there's no answers. (Family 3) |
| If there's an acknowledgement of the family, they get invited to a face to face, it shows there is a process in place whereby if you looked at it in the right light, people were trying to learn a lesson here. That there is process in place for learning that lesson. (Family 3) |
| The DHB was initially very defensive, which I kind of understand because their media liaison is suddenly getting absolutely hammered by every outlet you can imagine. But from a victim’s point of view to come out and straight off the bat say they did nothing wrong it's just a real slap in the face, at the time we didn’t know any better. In hindsight it set the pattern for the DHB’s response so as far as the process around dealing with the DHB. Initially it was all done through the media. (Family 3) |
| [Ideally] [the DHB] is going to be your point of contact, if you have any questions so this is now your liaison with us, we’re going to start a process, we’re going to review what happened here and try and ensure that this never happens again. Then go through that review process and then give the victims something they can review. And have a right of response to say we don’t understand this, can we have more information, or that sounds great or a right to question what happened. That would be great, and then it might give you a better sense of being involved and closure, because something has come out of the process. (Family 3) |
| From our perspective, there have been no learnings. Whether there has been an inquiry or internal reviews…we’ve never been informed or learnt anything about it. as far as [we’re] concerned, nothing’s happened, it's still back at where it was in the very beginning, where someone was killed and people were defensive on it and nothing ever came out about it. (Family 3) |
| You have to be giving that information to the public or giving that information at least to the victim’s family. So they go okay so it doesn’t fix it, there's still that loss, but something’s been done so that it may not happen again in the future. As much as [my family] wanting to have somebody to point the finger at, I think a big part of that healing process would have happened if there’d just been some acknowledgement that you had a loss…So your loss wasn’t in vain and we are acknowledging that you had a loss and that we are going to do something whatever that process may be, we’re going to do something about it. (Family 3) |
| It leaves me, with a blank space in this story. It’s as if this matter will never really be settled. And yet we have to get on with our lives. (Family 3) |
| When [the psychiatrist] sat down and he explained the reasoning and why they had come to that conclusion, and when I actually understood that I did get a form of peace with that. That actually meant a lot to me that day when the [the court] organised that. (Family 4) |
| I don’t believe that we should have had to have waited till then [the Coronial inquiry]to have been afforded that [humanity for the victim], it was a long time. It re-traumatised us in that sense because we had to live through everything again and stuff that we’d learnt over the years we’d had coping mechanisms and that to deal with. (Family 4) |
| The families need to be involved in that side of it [DHB inquiries]. At that time when the grief is really, really raw, I think that's actually a good time. Although it's probably isn’t for everybody, for us it would have helped. We could have dealt with it then and perhaps the lengthy process would have been shortened because we had to wait six and a half years by the time we got the Coroner’s report before that resolution. (Family 4) |
| The apology might have been nice first. I'm not sure, I'm still very angry with them, because we still don’t really have any answers on [the DHB] side. (Family 4) |
| I guess mostly just answers. But answers that probably should have been available to us and information that should have been there, the fact that we shouldn’t have had to go to so much effort to try and find out this stuff. Even to this day [the DHB] shut us down, even after everything that's come out of the Coroner’s inquest, they still are trying to cover things over. (Family 4) |
| [The DHB] could have eased our suffering by letting us know that someone was doing something. They didn’t need to wait for the Coroner’s report or the actual inquiry for them to [take action]…When they were questioned by the Coroner’s attorney and stuff hadn’t been put into place it was like they were lying all over again trying to just cover themselves up. Just be honest, if you haven’t done it say so, but don’t say we’ve done that and then get called out for it, because you look even more stupid. Just be honest and say, we are working on it. (Family 4) |
| It was horrendous. I think it’s [the case] got the record now for being one of the longest awaited Coronial appeals of reports. It was terrible. I think that they should have taken that into account on what they did to our mental health as well. Because for us, our lives had been on hold for [many] years until that date. (Family 4) |
| I don’t know whether in this case because the perpetrator was another mental health patient, whether that influenced the [case] in any way from the DHB side…we’ve got no idea whether there were question marks over the adequacy of his care. (Family 5) |
| There were things about the service that we said that's not good, this team not talking to that team and the whole process of getting [the victim] committed initially. The whole focus sort of switched to the justice side of things…I never felt like the DHB was remiss or I expected more from them. That email [DHB correspondence] was like, we haven’t heard anything about that review. (Family 5) |
| It would give you some sense that they acknowledged that we have unanswered questions and want to be informed. (Family 5) |
| I would really have liked to have had more of a follow-up [from the DHB] and found out what they found. At the time we didn’t chase it up…I think the emphasis should have come from them. (Family 5) |
| I suppose the trial filled a big void for us in terms of information. If he had just pleaded guilty, if there hadn’t been a trial, we wouldn’t have got all that information, it probably would have left us asking a whole lot of questions…. the trial was over and he got his sentence and so that felt like it was all completed. But it doesn’t feel like it's complete cos you’ve still got that expectation that you might get some feedback [from the DHB]. (Family 5) |
| It is in the back of your mind that you know you're not complete cos you haven’t had the findings [from the DHB inquiry]. (Family 5) |
| But for all we know there may be things in the review that we totally disagree with. We haven’t read it. [Would like] certainly the opportunity to comment on it and either agree or disagree with aspects of it. It seemed like [the victim] died and then that was it. I think if we’d got something back from the report, we would have got some sort of sense of closure. (Family 5) |
| If there were areas that could be improved and the DHB was aware of those and considered acting on them to improve the system, that would be comforting from our point of view to know that some good would come out - the service might improve because of it - and also give us a little bit of closure. (Family 5) |
